# Percutaneous coronary intervention in patients with cancer using bare metal stents compared to drug-eluting stents

**DOI:** 10.3389/fcvm.2022.901431

**Published:** 2022-10-19

**Authors:** Talha Ahmed, Homam Moussa Pacha, Antoine Addoumieh, Efstratios Koutroumpakis, Juhee Song, Konstantinos Charitakis, Konstantinos Dean Boudoulas, Mehmet Cilingiroglu, Konstantinos Marmagkiolis, Cindy Grines, Cezar A. Iliescu

**Affiliations:** ^1^Division of Cardiovascular Medicine, University of Texas Health Science Center at Houston, Houston, TX, United States; ^2^Division of Cardiovascular Medicine, MD Anderson Cancer Center, University of Texas, Houston, TX, United States; ^3^Department of Biostatistics, MD Anderson Cancer Center, University of Texas, Houston, TX, United States; ^4^Division of Cardiovascular Medicine, The Ohio State University, Columbus, OH, United States; ^5^University of South Florida, Tampa, FL, United States; ^6^Division of Cardiovascular Medicine, Northside Hospital Cardiovascular Institute, Atlanta, GA, United States

**Keywords:** percutaneous coronary intervention, bare metal stents, drug-eluting stents, cardio-oncology, revascularization

## Abstract

**Background:**

Management of coronary artery disease (CAD) is unique and challenging in cancer patients. However, little is known about the outcomes of using BMS or DES in these patients. This study aimed to compare the outcomes of percutaneous coronary intervention (PCI) in cancer patients who were treated with bare metal stents (BMS) vs. drug-eluting stents (DES).

**Methods:**

We identified cancer patients who underwent PCI using BMS or DES between 2013 and 2020. Outcomes of interest were overall survival (OS) and the number of revascularizations. The Kaplan–Meier method was used to estimate the survival probability. Multivariate Cox regression models were utilized to compare OS between BMS and DES.

**Results:**

We included 346 cancer patients who underwent PCI with a median follow-up of 34.1 months (95% CI, 28.4–38.7). Among these, 42 patients were treated with BMS (12.1%) and 304 with DES (87.9%). Age and gender were similar between the BMS and DES groups (*p* = 0.09 and 0.93, respectively). DES use was more frequent in the white race, while black patients had more BMS (*p* = 0.03). The use of DES was more common in patients with NSTEMI (*p* = 0.03). The median survival was 46 months (95% CI, 34–66). There was no significant difference in the number of revascularizations between the BMS and DES groups (*p* = 0.43). There was no significant difference in OS between the BMS and DES groups in multivariate analysis (*p* = 0.26). In addition, independent predictors for worse survival included age > 65 years, BMI ≤ 25 g/m^2^, hemoglobin level ≤ 12 g/dL, and initial presentation with NSTEMI.

**Conclusions:**

In our study, several revascularizations and survival were similar between cancer patients with CAD treated with BMS and DES. This finding suggests that DES use is not associated with an increased risk for stent thrombosis, and as cancer survival improves, there may be a more significant role for DES.

## Introduction

Cardiovascular disease and cancer frequently coexist in an increasingly aging population and share the same risk factors (1). They are also the leading causes of death in developed countries, accounting for two-thirds of disease-related mortality (1). Despite the increased prevalence of thrombocytopenia and bleeding tendencies, cancer is often associated with a hypercoagulable state with increased platelet activation and aggregation. In addition, many chemotherapeutic agents are associated with angina, myocardial infarction (MI), and acceleration of pre-existing coronary artery disease (CAD), while radiotherapy is associated with CAD through direct endothelial injury (2–4). All the aforementioned factors make the management of cancer patients' CAD unique as well as challenging.

A history of cancer is independently associated with an increased risk of major adverse cardiovascular events (MACE) (5–7). The current generation of drug-eluting stents (DES) has been proven to reduce the risk of restenosis and stent thrombosis compared to bare-metal stents (BMS). However, data suggesting the preference of DES over BMS in the cancer population are lacking. The perceived need for a shorter course of dual antiplatelet treatment (DAPT) used to make the use of BMS an attractive alternative, particularly in patients with increased bleeding risk and an expectant need for cancer-directed surgery and/or procedures (8, 9).

There are limited data on the outcomes of cancer patients requiring PCI when directly comparing BMS with DES. Additionally, most randomized controlled trials making such comparisons exclude patients with active malignancy and treatment. With a growing number of patients with cancer, it is essential to study the outcomes of different types of stents and the duration of antiplatelet agents (10). The current study examines clinical and procedural characteristics and clinical outcomes in cancer patients with CAD treated with BMS vs. DES.

## Materials and methods

### Patient population

This was a single-center, retrospective, observational study approved by the MD Anderson Cancer Center Institutional Review Board. The requirement to obtain informed consent was waived, and the data were deidentified. All cancer patients who underwent PCI between January 2013 and December 2020 were included. Patients were further divided into two groups based on the type of intervention performed using either BMS or DES. Patients treated with balloon angioplasty alone were excluded. The decision to treat a patient with either of these strategies was based on the clinical characteristics of the individual patient. It was left to the discretion of the treating physicians.

Patient characteristics were collected using electronic medical records, including age, sex, race, body mass index, indication for primary PCI, comorbidities (history of diabetes mellitus, hypertension, hyperlipidemia, end-stage renal disease, peripheral vascular disease, stroke, or transient ischemic attack, previous coronary artery bypass graft, and PCI, etc.), as well as laboratory variables (hemoglobin, platelet count, creatinine, lipid panel, troponin, and B type natriuretic peptide/BNP, etc.), type of malignancy (solid vs. hematological), and intracoronary imaging used (intravascular ultrasound/IVUS and optical coherence tomography/OCT), as MD Anderson Catheterization laboratory is not an ST-elevation myocardial infarction (STEMI) receiving center, so these patients were not included. All other indications of revascularizations include “cardiomyopathy,” “positive stress test,” “unstable angina,” “non STEMI,” and “angina with prior history of CAD.” Propensity score matching was conducted to select patients treated with BMS and comparable patients treated with DES. Furthermore, information related to primary outcomes was collected. The term “number of revascularizations” was defined by the total number of revascularizations needed for either the target vessel stented with either BMS or DES during the index procedure or for other arteries.

### Outcomes

The primary endpoints included all-cause mortality and the number of revascularizations at the end of the follow-up period, while the secondary outcome was cardiovascular death.

### Statistical analysis

Continuous variables were described as means ± standard deviations (SDs) or medians with interquartile ranges (IQRs). As appropriate, categorical variables were described as counts and percentages. Patient characteristics were compared between BMS and DES by a two-sample *t*-test or Wilcoxon rank-sum test for continuous variables and a Chi-square test or Fisher's exact test for categorical variables. Overall, survival time was defined as the interval between index PCI intervention and death. It was determined at the last follow-up if the patient was alive during the follow-up. The Kaplan–Meier method was used to estimate the survival probability. Univariate and multivariate Cox regression models were used to compare BMS and DES overall survival. The multivariate logistic regression model initially included covariates with a significant or marginally significant *p*-value based on univariate logistic regression analysis. The stepwise selection method was then utilized to include significant variables in the multivariate model. The propensity score, the predicted probability of receiving BMS, was calculated using a multivariate logistic regression model including significant factors. 1:1 propensity score matching and a 1:2 propensity score matching were conducted to select patients treated with BMS and comparable patients treated with DES using a one-to-many match macro using a greedy algorithm. A univariate Cox regression model was utilized to compare overall survival between BMS and DES in propensity score-matched cohorts. A *p* < 0.05 indicates statistical significance. For data analysis, SAS version 9.4 (SAS Institute, Inc., Cary, North Carolina) was used.

## Results

### Baseline characteristics

**Figure 1:**
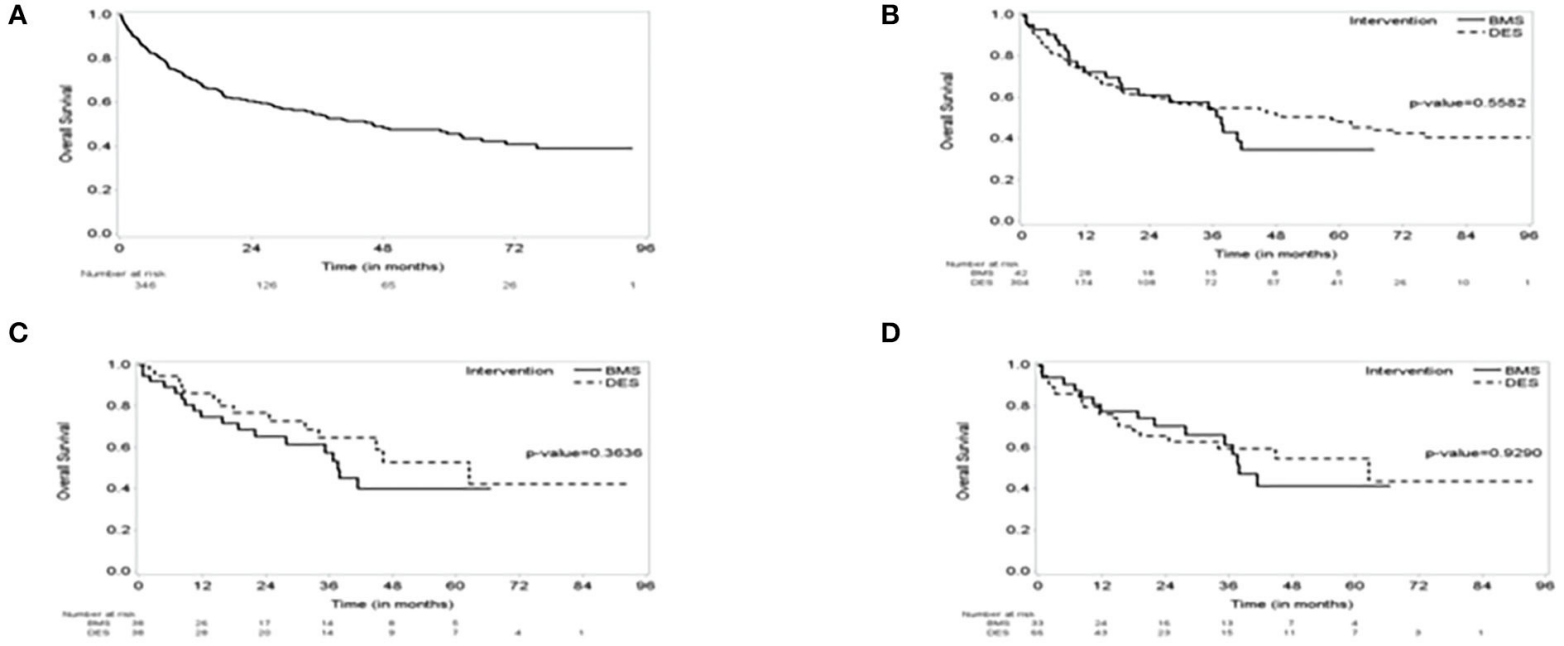
The Kaplan–Meier (KM) survival curve. **(A)** The KM curve of the entire group showing Median survival: 46 months (95% CI, 34–66); median follow-up of 34 months (95% CI, 28–39). **(B)** KM Survival curve by intervention showing no difference in survival over the follow-up period between bare metal stent (BMS) vs. drug-eluting stent (DES). **(C)** a 1:1 propensity score-matched cohorts of BMS vs. DES showing no difference in survival. **(D)** a 1:2 propensity score-matched cohorts of BMS vs. DES showing no difference in survival.

**Figure 2:**
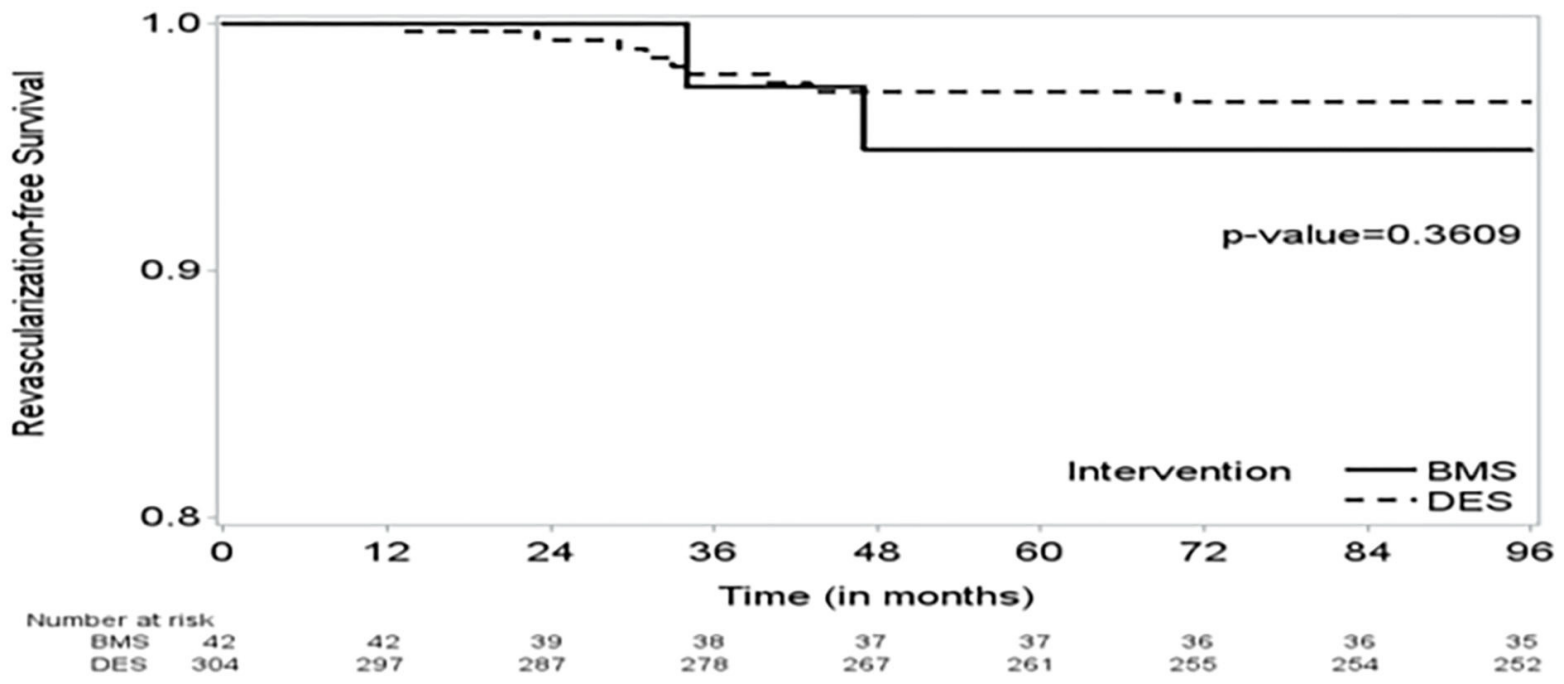
The Kaplan–Meier (KM) curve on time to revascularization shows no significant difference in time to revascularization between the BMS and DES groups.

The study included 346 CAD cancer patients treated with BMS (*n* = 42) or DES (*n* = 304), while patients treated with POBA (*n* = 9) were excluded (Table 1). The median follow-up time, estimated by the reverse of the Kaplan–Meier method, was 34.1 months (95% CI, 28.4–38.7). The median survival time was 46.2 months (95% CI, 34.0–66.0) (Figures 1, 2). Patient characteristics of the intervention (BMS vs. DES) are summarized in Table 1. Some variables showed significant differences between the BMS and DES groups: BMS was more prevalent in blacks, while DES was more commonly seen in whites. Lipid panels, including cholesterol and mean LDL, were higher in the BMS group. The BMS group had a higher prevalence of family history of premature CAD, while those treated with DES had a significantly increased number of prior MI and PCI. DES use was more common in patients with non-ST segment elevation MI (NSTEMI).

**Table 1:** Descriptive statistics by the intervention (BMS vs. DES).

**Variable**	**BMS (*n* = 42)**	**DES (*n* = 304)**	***P*-value^a^**
Age (years)^b^	70.04 ± 9.79	67.16 ± 10.27	0.0870^c^
Number of revascularizations^d^	0 (0–0)	0 (0–0)	0.4263
Platelet count (10^3^/uL)^d^	178 (158–246)	188 (138–253)	0.7883
Absolute Neutrophil Count (10^3^/uL)^d^	3.9 (2.7–5.9)	4.44 (3–6)	0.5012
INR^d^	1.1 (1.02–1.27)	1.1 (1.01–1.2)	0.4705
Creatinine (mg/dL)^d^	1.08 (0.8–1.3)	1.03 (0.84–1.24)	0.7664
Hemoglobin (g/dL)^b^	11.89 ± 2.32	11.65 ± 2.13	0.4985^c^
Triglyceride (mg/dL)^d^	131 (80–224)	127 (88–170)	0.9536
Cholesterol (mg/dL)^d^	163 (145–224)	143 (114–171)	0.0243
HDL (mg/dL)^c^	45.00 ± 15.03	40.98 ± 13.93	0.2678^c^
LDL (mg/dL)^d^	91.5 (65.5–146)	73 (48–99)	0.0462
VLD (mg/dL)^d^	31 (16–45)	23.5 (17–34)	0.6332
BNP (pg/mL)^d^	347.5 (100–785.5)	422 (165–644)	0.6398
Troponin (ng/mL)^d^	0.66 (0.03–5.1)	0.4 (0.03–2.4)	0.5883
BMI (kg/m^2^)^b^	28.82 ± 6.06	28.91 ± 6.09	0.9329^c^
**Gender**			
Male	33 (78.6%)	237 (78%)	0.9286^e^
Female	9 (21.4%)	67 (22%)	
**Race**			
White	20 (47.6%)	206 (68.4%)	0.0282^e^
Black	5 (11.9%)	23 (7.6%)	
Other	17 (40.5%)	72 (23.9%)	
**Number of revascularization**			
0	39 (92.9%)	271 (89.1%)	0.8719^f^
1	3 (7.1%)	20 (6.6%)	
2	0 (0.0%)	10 (3.3%)	
3	0 (0.0%)	3 (1%)	
**Intracoronary imaging**			
None	16 (38.1%)	118 (38.8%)	0.5256^e^
IVUS	24 (57.1%)	156 (51.3%)	
OCT	2 (4.8%)	30 (9.9%)	
**Cancer type**			
Solid	34 (81%)	198 (71.2%)	0.1881^e^
Hematological	8 (19%)	80 (28.8%)	
Smoker ≥1 years	24 (58.5%)	182 (69.3%)	0.5087^e^
Hypertension	38 (92.7%)	264 (91.7%)	1.0000^f^
Dyslipidemia	31 (77.5%)	232 (82%)	0.4954^e^
Family History Premature CAD	13 (34.2%)	34 (11.8%)	0.0002^e^
Prior MI	8 (21.1%)	103 (37.7%)	0.0444^e^
Prior Heart Failure	8 (20.5%)	65 (25.4%)	0.5108^e^
Peripheral Artery Disease	3 (7.9%)	42 (16.6%)	0.1663^e^
Chronic Lung Disease	3 (7.9%)	44 (17.4%)	0.1380^e^
Diabetes	11 (28.9%)	128 (48.7%)	0.0226^e^
Prior PCI	2 (11.1%)	66 (35.1%)	0.0386^e^
Prior CABG	0 (0.0%)	24 (13.4%)	0.1361^f^
**Indication for Revascularization**			
Cardiomyopathy	6 (14.3%)	43 (14.1%)	0.9804^e^
Abnormal Stress test	16 (38.1%)	73 (24%)	0.0503^e^
Stable CAD	16 (38.1%)	130 (42.8%)	0.5659^e^
Unstable Angina	7 (16.7%)	71 (23.4%)	0.3309^e^
NSTEMI	5 (11.9%)	82 (27%)	0.0323^e^

BMI, Body mass index; IVUS, Intravascular ultrasound; OCT, optical coherence tomography; MI, myocardial Infarction; CAD, coronary artery disease; PCI, Percutaneous coronary intervention; CABG, coronary artery bypass grafting; NSTEMI, non-ST elevation MI.

^a^Wilcoxon rank-sum test was used unless specified, ^b^Mean ± SD are presented, ^c^ Two sample t-test was used, ^d^Median (IQR) are presented, ^e^Chi-square test was used, and ^f^Fisher's exact test was used. P value < 0.05 suggesting statistical significance.

### Univariate Cox analysis results

Univariate analysis results are presented in Table 2. Age, higher INR, lower hemoglobin, lower body mass index (BMI), absence of hypertension, and primary PCI indication of NSTEMI were significantly associated with an increased risk of death.

**Table 2:** Univariate Cox model (on overall survival time); with 346 who performed BMS or DES (147 deaths).

**Variable**	**HR (95% CI)**	***P*-value**
Age (years)^a^	1.031 (1.013–1.049)	0.0006
Platelet count (10^3^/uL)^a^	0.999 (0.997–1.001)	0.3660
Absolute Neutrophil Count (10^3^/uL)^a^	1.029 (0.992–1.068)	0.1273
INR	0.984 (0.940–1.031)	0.5088
Creatinine (mg/dL)^a^	1.002 (0.981–1.022)	0.8784
Hemoglobin (g/dL) ^a^	0.800 (0.737–0.869)	< 0.0001
Triglyceride (mg/dL)^a^	0.999 (0.996–1.002)	0.4313
Cholesterol (mg/dL)^a^	0.998 (0.994–1.003)	0.4079
HDL (mg/dL)^a^	0.990 (0.974–1.006)	0.2084
LDL (mg/dL)^a^	0.999 (0.994–1.004)	0.7545
VLD (mg/dL)^a^	1.000 (0.982–1.018)	0.9775
BNP (pg/mL)^a^	1.000 (1.000–1.000)	0.9188
Troponin (ng/mL)^a^	0.984 (0.953–1.017)	0.3335
BMI (kg/m^2^)^a^	0.943 (0.915–0.972)	0.0002
**Gender**		
Male	1.000	
Female	1.071 (0.720–1.592)	0.7344
**Race**		
White	1.000	
Black	1.442 (0.836–2.488)	0.1880
Other	0.824 (0.553–1.229)	0.3424
**Intervention group**		
BMS	1.000	
DES	0.873 (0.554–1.375)	0.5585
**Intracoronary imaging**		
IVUS	1.000	
None	1.130 (0.802–1.592)	0.4838
OCT	1.036 (0.575–1.865)	0.9072
**Cancer type**		
Solid	1.000	
Hematological	0.824 (0.572–1.187)	0.2990
Smoker≥1 year^b^	1.181 (0.838–1.665)	0.3408
Hypertension^b^	0.594 (0.358–0.987)	0.0443
Dyslipidemia^b^	1.095 (0.710–1.689)	0.6816
Family History Premature CAD^b^	1.439 (0.932–2.223)	0.1008
Prior MI^b^	0.760 (0.523–1.103)	0.1486
Prior Heart Failure^b^	1.350 (0.918–1.986)	0.1270
Peripheral Artery Disease^b^	1.215 (0.767–1.923)	0.4063
Chronic Lung Disease^b^	1.493 (0.974–2.289)	0.0661
Diabetes^b^	0.942 (0.661–1.341)	0.7383
Prior PCI^b^	1.063 (0.666–1.697)	0.7969
Prior CABG^b^	0.975 (0.484–1.962)	0.9430
**Indication for Revascularization**		
Cardiomyopathy^b^	1.092 (0.699–1.705)	0.6989
Abnormal Stress test^b^	0.707 (0.482–1.038)	0.0766
Stable CAD^b^	1.072 (0.775–1.484)	0.6739
Unstable Angina^b^	0.718 (0.473–1.091)	0.1203
NSTEMI^b^	1.961 (1.386–2.774)	0.0001

The number of revascularization is not included in this analysis as this variable is not a baseline characteristic. (i.e., patients with revascularization are likely to have longer survival as the survival time is calculated from initial vascularization and patients need to be survived to be revascularized).

BMI, Body mass index; IVUS, Intravascular ultrasound; OCT, Optical coherence tomography; MI, Myocardial Infarction; CAD, Coronary artery disease; PCI, Percutaneous coronary intervention; CABG, Coronary artery bypass grafting; NSTEMI, Non ST-elevation MI.

a HR in 1 unit change is presented along with 95% CI.

b HR considering no group as a reference, is presented along with 95% CI. P value < 0.05 suggesting statistical significance.

### Multivariate Cox analysis results

A multivariate Cox model initially considered the age at intervention, INR group, hemoglobin group, family history of premature CAD, chronic lung disease, BMI group, and an indication of primary PCI (abnormal stress test or NSTEMI). Age, hemoglobin, BMI, and indication of NSTEMI remained significant in multivariate analysis. Therefore, multivariate models, including age group, hemoglobin group (using 12 g/dl as a cutoff value), BMI group, and an indication of NSTEMI, are presented in Table 3. After adjusting for age, hemoglobin, BMI, and indication of NSTEMI, BMS and DES did not show a significant difference in overall survival.

**Table 3:** Multivariate Cox model (on overall survival time).

**Variable**	**Level**	**HR (95% CI)**	***P*-value**
Age group	≤ 65 years	1.000	
	>65 years	1.592 (1.087–2.334)	0.0170
Hemoglobin group	≤ 12 g/dL	1.000	
	>12 g/dL	0.481 (0.328–0.706)	0.0002
Intervention group	BMS	1.000	
	DES	0.763 (0.479–1.216)	0.2561
BMI group	≤ 25 g/m2	1.000	
	25–30 g/m2	0.811 (0.541–1.216)	0.3102
	>30 g/m2	0.585 (0.378–0.906)	0.0163
Indication: NSTEMI	No	1.000	
	Yes	1.629 (1.110–2.391)	0.0127

Including 323 patients with either BMS or DES considering age, hemoglobin (12 g/dL as a cutoff value), intervention, BMI, and an indication of NSTEMI.

### Propensity score matching

Some patients were treated with BMS, while others with DES, and these interventions were not randomly allocated. To make a fair comparison between BMS and DES in outcomes, we calculated the propensity score using a logistic regression model to predict being treated with BMS. The logistic regression model initially considered significant or marginally significant variables in univariate logistic regression models (age at intervention, family history of premature CAD, race, prior MI, and diabetes). The stepwise selection method selected family history of premature CAD, race, and diabetes in the final multivariate logistic regression model. Using this model, we calculated the propensity score as the predicted probability of receiving BMS for given covariates. Using these propensity scores, we selected a 1:1 propensity score-matched cohorts (38 BMS vs. 38 DES) and a 1:2 propensity score-matched cohorts (33 BMS vs. 66 DES). In these cohorts, BMS and DES did not show significant differences in overall survival (Tables 4a,b).

**Table 4:** Propensity score matching.

		**Univariate Cox model**		**Multivariate Cox model**	
**Variable**	**Level**	**HR (95% CI)**	***P*-value**	**HR (95% CI)**	***P*-value**
**(a) 1:1 matching for BMS to DES (38 BMS vs. 38 DES were chosen)**					
Intervention group	BMS	1.000		1.000	
	DES	0.724 (0.360–1.457)	0.3657	0.739 (0.367–1.489)	0.3974
Hemoglobin group	≤ 12 g/dL			1.000	
	>12 g/dL			0.516 (0.257–1.036)	0.0629
		**Univariate Cox model**		**Multivariate Cox model**	
**Variable**	**Level**	**HR (95% CI)**	***P*** **-value**	**HR (95% CI)**	***P*** **-value**
**(b) 1:2 matching for BMS to DES (33 BMS vs. 66 DES were chosen)**					
Intervention group	BMS	1.000		1.000	
	DES	0.971 (0.508–1.856)	0.9287	0.941 (0.489–1.809)	0.8545
Hemoglobin group	≤ 12 g/dL			1.000	
	>12 g/dL			0.517 (0.271–0.988)	0.0460

The “number of revascularizations” was compared between the two groups: BMS vs. DES (Table 5a). Propensity score matching was also performed for the “number of revascularizations” (Tables 5b,c).

**Table 5:** Analysis of the number of revascularizations between BMS vs. DES group, including 1:1 and 1:2 propensity-matched analysis.

**Covariate**	**Levels**	**BMS (*n* = 42)**	**DES (*n* = 304)**	***P*-value**
**(a) Including all patients with BMS or DES**				
Number of revascularization	Median (Q1-Q3)	0 (0–0)	0 (0–0)	0.4263
	Mean ± SD	0.07 ± 0.26	0.16 ± 0.51	0.0745
Number of revascularization	0	39 (92.9%)	271 (89.1%)	0.8719
	1	3 (7.1%)	20 (6.6%)	
	2	0 (0%)	10 (3.3%)	
	3	0 (0%)	3 (1%)	
**Covariate**	**Levels**	**BMS (*****n*** = **38)**	**DES (*****n*** = **38)**	***P*** **-value**
**(b) 1:1 Propensity score matched cohorts**				
Number of revascularization	Median (Q1-Q3)	0 (0–0)	0 (0–0)	0.6560
	Mean (SD)	0.08 ± 0.27	0.16 ± 0.49	0.3927
Number of revascularization	0	35 (92.1%)	34 (89.5%)	0.6745
	1	3 (7.9%)	2 (5.3%)	
	2	0 (0%)	2 (5.3%)	
**Covariate**	**Levels**	**BMS (*****n*** = **33)**	**DES (*****n*** = **66)**	***P*** **-value**
**(c) 1:2 propensity score matched cohorts**				
Number of revascularization	Median (Q1-Q3)	0 (0–0)	0 (0–0)	0.6144
	Mean (SD)	0.09 ± 0.29	0.17 ± 0.48	0.3353
Number of revascularization	0	30 (90.9%)	58 (87.9%)	0.7447
	1	3 (9.1%)	5 (7.6%)	
	2	0 (0%)	3 (4.5%)	

### Secondary outcomes and other statistical analysis

**Figure 3:**
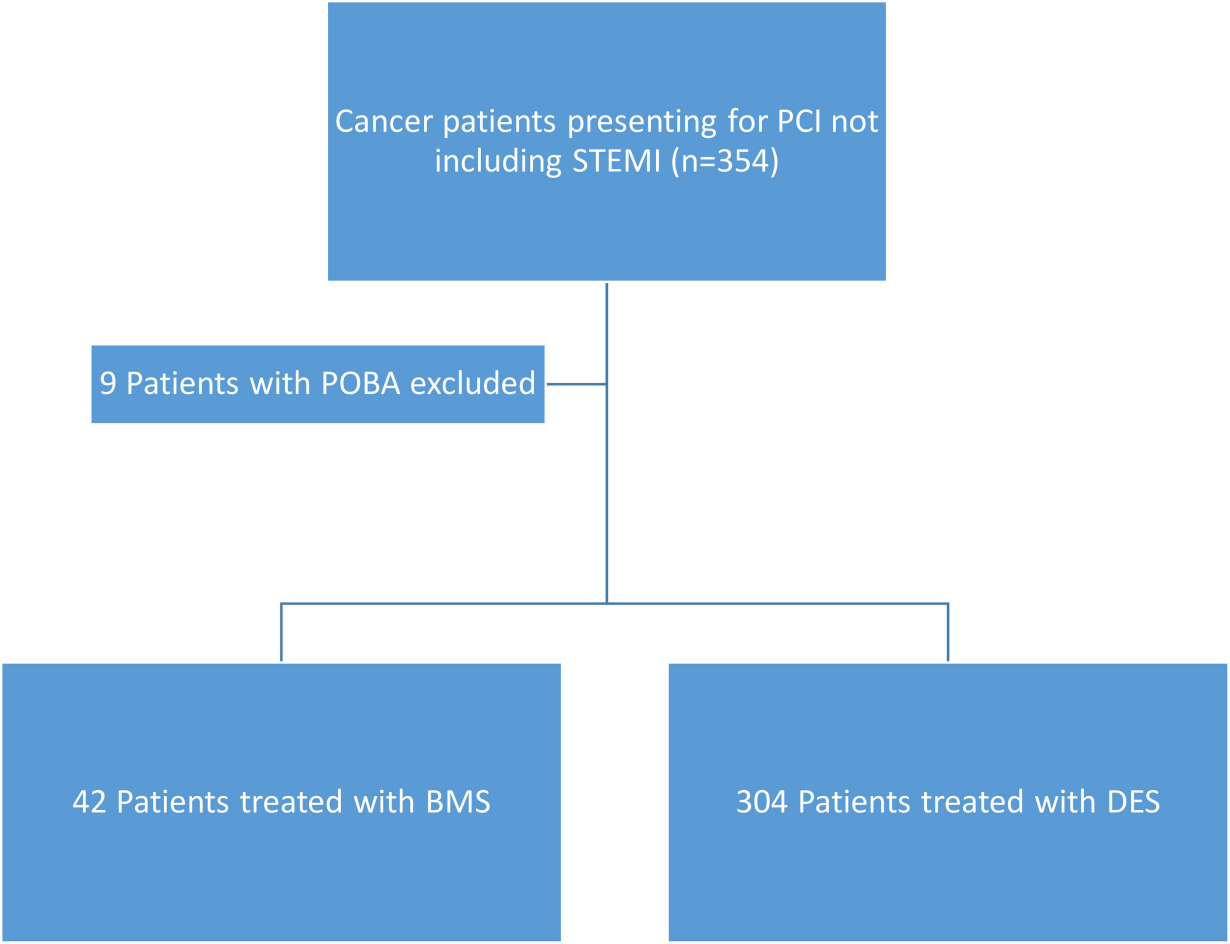
Patient flow chart showing inclusion criteria for the patients. STEMI, ST-elevation myocardial infarction; POBA, plain old balloon angioplasty; BMS, bare metal stents; DES, drug-eluting stents.

Univariate Fine-Gray models, considering cardiovascular-specific death as an event of interest and death as a competing risk event, revealed no significant difference in cardiovascular outcomes between BMS vs. DES (Table 6). Detailed cancer characteristics for patients in the BMS and DES groups are provided in Table 7. The number of patients who underwent BMS and DES each year during the study duration (2013–2020) is provided in Table 8. A descriptive patient flowchart for inclusion in the study is provided in Figure 3.

**Table 6:** Cardiovascular specific survival: Univariate Fine-Gray models, considering cardiovascular specific death as an event of interest and death as a competing risk event.

**Variable**	**HR (95% CI)**	***P*-value**
Age (years)	1.034 (0.970–1.104)	0.3064
Platelet count (10^3^/uL)	1.000 (0.997–1.003)	0.9275
Absolute Neutrophil Count (10^3^/uL)	1.056 (1.019–1.095)	0.0030
INR	1.002 (0.976–1.028)	0.8864
Creatinine (mg/dL)	0.995 (0.977–1.012)	0.5368
Hemoglobin (g/dL)	0.874 (0.716–1.066)	0.1848
Triglyceride (mg/dL)	1.003 (0.998–1.007)	0.2097
Cholesterol (mg/dL)	0.989 (0.978–0.999)	0.0354
HDL (mg/dL)	0.982 (0.942–1.024)	0.3983
LDL (mg/dL)	0.978 (0.963–0.994)	0.0071
VLD (mg/dL)	1.025 (0.998–1.054)	0.0682
BNP (pg/mL)	1.000 (0.999–1.000)	0.5436
Troponin (ng/mL)	0.866 (0.730–1.028)	0.0995
BMI (kg/m^2^)	0.955 (0.907–1.005)	0.0765
**Gender**		
Female	1.000	
Male	1.443 (0.498–4.181)	0.4996
**Race**		
White	1.000	
Black	1.572 (0.462–5.344)	0.4687
Other	0.600 (0.201–1.787)	0.3588
**Intervention group**		
BMS	1.000	
DES	3.394 (0.464–24.830)	0.2288
**Intracoronary imaging**		
IVUS	1.000	
None	1.455 (0.633–3.341)	0.3770
OCT	1.040 (0.240–4.506)	0.9582
**Cancer type**		
Solid	1.000	
Hematological	0.839 (0.347–2.026)	0.6960
**Indication for Revascularization**		
Cardiomyopathy	1.618 (0.610–4.290)	0.3336
Abnormal Stress test	0.243 (0.059–1.008)	0.0512
Stable CAD	0.947 (0.422–2.125)	0.8957
Unstable Angina	1.144 (0.457–2.861)	0.7743
NSTEMI	2.232 (0.995–5.005)	0.0515

**Table 7:** Number of patients with BMS vs. DES per year (2013–2020).

**Frequency**	**BMS** ***n* (%)**	**DES** ***n* (%)**
2013	0 (0)	38 (100)
2014	4 (9.5)	38 (90.5)
2015	18 (39)	28 (61)
2016	12 (21.4)	44 (78.6)
2017	5 (11.6)	38 (88.4)
2018	2 (4.1)	47 (95.9)
2019	0 (0)	49 (100)
2020	1 (4.3)	22 (95.7)

**Table 8:** Cancer characteristics by intervention.

**Covariate**	**Levels**	**BMS**	**DES**	***P*-value**
Cancer type	Solid	34 (81%)	198 (71.2%)	0.1881
	Hematologic	8 (19%)	80 (28.8%)	
Primary Cancer Type	1 Leukemia	7 (16.7%)	32 (11.6%)	0.0075
	2 Myeloma	1 (2.4%)	21 (7.6%)	
	3 Lymphoma	0 (0%)	26 (9.4%)	
	4 Lung	1 (2.4%)	41 (14.8%)	
	5 Colon/rectal	4 (9.5%)	15 (5.4%)	
	6 Breast	2 (4.8%)	11 (4%)	
	7 Pancreatic	3 (7.1%)	6 (2.2%)	
	8 Uterine	0 (0%)	2 (0.7%)	
	9 Ovarian/Endometrial	0 (0%)	3 (1.1%)	
	11 Prostate	1 (2.4%)	19 (6.9%)	
	12 Skin	2 (4.8%)	5 (1.8%)	
	13 Melanoma	4 (9.5%)	8 (2.9%)	
	14 Stomach/Esophageal	1 (2.4%)	12 (4.3%)	
	15 Renal/bladder	3 (7.1%)	31 (11.2%)	
	16 Other	3 (7.1%)	9 (3.2%)	
	17 Thyroid	1 (2.4%)	8 (2.9%)	
	18 ENT	4 (9.5%)	15 (5.4%)	
	19 Neurological	1 (2.4%)	2 (0.7%)	
	20 Liver	4 (9.5%)	8 (2.9%)	
	21 Endocrine	0 (0%)	3 (1.1%)	
Primary cancer group	1 Leukemia	7 (16.7%)	32 (11.6%)	0.0049
	2 Myeloma	1 (2.4%)	21 (7.6%)	
	3 Lymphoma	0 (0%)	26 (9.4%)	
	4 Lung	1 (2.4%)	41 (14.8%)	
	5 GI	12 (28.6%)	40 (14.4%)	
	6 Breast	2 (4.8%)	10 (3.6%)	
	7 Gynecological	0 (0%)	5 (1.8%)	
	8 Prostate/Testicular	1 (2.4%)	22 (7.9%)	
	9 Skin	6 (14.3%)	13 (4.7%)	
	10 Renal/bladder	3 (7.1%)	28 (10.1%)	
	11 Other	4 (9.5%)	12 (4.3%)	
	12 Endocrine	1 (2.4%)	9 (3.2%)	
	13 ENT	4 (9.5%)	18 (6.5%)	
Prior Chemotherapy	0	8 (33.3%)	61 (31.1%)	0.8256
	1	16 (66.7%)	135 (68.9%)	
Prior radiation	0	15 (62.5%)	118 (59.9%)	0.8058
	1	9 (37.5%)	79 (40.1%)	
Active Chemotherapy	0	14 (58.3%)	116 (58.3%)	0.9969
	1	10 (41.7%)	83 (41.7%)	

## Discussion

Our study showed that 1) the number of revascularizations (including target and other vessels) in cancer patients with CAD treated with BMS vs. DES was similar during the follow-up period, and 2) the all-cause mortality between BMS and DES did not differ significantly. These are important findings since cancer and cardiovascular disease are the most prevalent diseases worldwide. Data on outcomes after percutaneous intervention in these patients are scant, and the evidence-based treatment regimen for CAD in this group of patients is not well established (11–13).

Several comorbid conditions affect patients with cancer, which influence their treatment in the setting of PCI. While cancer and its treatment can predispose patients to bleeding tendencies and thrombocytopenia, neoplasia by itself is a pro-coagulant state (14). This poses a unique challenge and highlights the need to evaluate thrombosis and bleeding risks carefully. In the setting of PCI, this information has a tremendous impact on the options for stenting and antiplatelet therapy (14). Several clinical studies have proven the superiority of DES over BMS in reducing the risk of restenosis and stent thrombosis compared with bare-metal stents (BMS) in non-cancer high-risk patients (9). In-stent restenosis, although of concern, may not be significant due to shorter-term survivorship from cancer. As cancer survival rates keep improving, the role of DES in improved restenosis becomes more important. A study using OCT to evaluate stent healing after DES placement showed adequate stent healing in cancer patients despite a shorter course of DAPT (< 6 months) in 61% of them. Findings were matched with stent healing value for DES in non-cancer patients (15).

Another concern for DES use in cancer patients is stent thrombosis, given the need for a shorter course of antiplatelet therapy in selected cases (16). In our study, the number of revascularizations was similar between the DES and BMS groups. Hence, DES is likely not associated with increased thrombotic risk in the cancer patient population. The idea of abbreviated DAPT after BMS appeals to the high-risk group of cancer patients (17). With recent advancements, the current-generation DES now possesses a reduced stent strut thickness and a unique drug fast-release profile that results in less powerful inhibition of intimal hyperplasia and rapid reendothelialization of stent struts. Given these qualities, a shorter duration of DAPT seems more feasible (18).

Recently published trials showed that 1 month of DAPT after PCI followed by aspirin monotherapy was non-inferior to 6 or 12 months of full antiplatelet therapy (18, 19). Interestingly, there was no difference in the occurrence of major bleeding and stent thrombosis between both groups. Similar studies are needed in a cancer population. Currently, the latest American College of Cardiology/American Heart Association (ACC/AHA) guidelines and European Society of Cardiology (ESC) guidelines still emphasize a class I recommendation for at least 6 months of DAPT in non-ACS for DES and 1 month for BMS, and 12 months of DAPT in ACS settings for both DES and BMS (20, 21). According to the ACC/AHA guidelines, discontinuation of aspirin may be considered 1–3 months after DES implantation with continued P2Y12 monotherapy in both stable ischemic heart disease (SIHD) and ACS patients (class 2a recommendation) (20). We believe future guidelines will continue to implement shorter courses of DAPT as more data supporting this becomes available, especially with advanced technology in stent development. This will favor DES use in such a high-risk cancer population.

Another important consideration is the increased requirement for anticoagulation in cancer patients due to their higher propensity for thrombosis and atrial fibrillation. The management of triple therapy in these patients poses its own challenges due to the high risk of bleeding and a decision regarding the timing of re-initiation of chemotherapy (22, 23). A recent large study on a national database suggested superior outcomes in patients with cancer with a DES placed compared with those with a bare-metal stent (BMS) placed (8). However, this was driven by higher in-hospital mortality and increased bleeding events in the BMS group, signifying a selection bias to use BMS for sicker patients requiring early discontinuation of DAPT for various reasons, including initiation of cancer therapy due to advanced disease (24, 25). Although the choice of a stent in our study was at the treating physician's discretion after shared decision-making with the patient, a key difference in baseline characteristics between the two groups was an increased number of patients with NSTEMI in the DES group.

A significant interplay exists between cancer and CAD. Given a high bleeding risk in patients with cancer, shorter-duration DAPT and BMS were historically preferred in the setting of percutaneous coronary intervention. However, factors such as chronic inflammation and chemotherapy/radiation-induced cardiotoxicity increase the risk of stent thrombosis and in-stent restenosis. Another important observation from this study is that in cancer patients, despite the increased inflammatory and prothrombotic state, the use of DES was not associated with a need for more revascularizations as compared to BMS. In a recent Italian registry, the use of BMS was extremely low, at 0.3 %, with the main reasons for BMS use being advanced age, ST-elevation myocardial infarction (STEMI), and physicians' perception of a high risk of bleeding (25).

Moreover, recent evidence from multiple studies suggests that shorter-duration DAPT is feasible with newer-generation DES and that percutaneous coronary intervention outcomes with the current generation of DES are better than with BMS (26, 27). Although the utilization of these stents in cancer patients is yet to be tested, in light of the current evidence, there is no reason for using BMS in any situation except for some cost-effectiveness. Moreover, the revolution of BMS vs. DES in our study indicates a stronger preference for using DES in the later years, with improvement in the design and generations of these stents.

Recent data suggest that routine use of intracoronary imaging leads to superior outcomes, which is paramount when shorter durations of DAPT are required (28–30). In our study, > 50% of the patients in either arm had IVUS as a part of their intervention, while almost 5% in BMS and 10% in DES underwent OCT. This highlights the role of optimizing PCI in this patient population, particularly given the increased likelihood that a shorter duration of DAPT may be required. This approach can avoid stent under-sizing and malapposition and residual untreated complications such as edge dissections, all of which may lead to worse outcomes, especially with a shorter duration of DAPT (13). When possible, bifurcation and overlapping stents should be avoided to reduce the risk of stent thrombosis (13).

## Study limitations

Our study included a large cohort of patients with cancer patients undergoing PCI with DES vs. BMS reported to date. However, it was a single-center retrospective observational study with known limitations, including relatively small sample size. Also, mortality data may be underestimated because we rely on our electronic medical records. Furthermore, the successful continuation of DAPT therapy in both arms could not be accurately confirmed due to the study's retrospective nature. Moreover, our study did not use the newest generations of stents, including zatarolimus-coated stents, polymer-free stents, nano-coated stents, etc., requiring shorter-term DAPT therapy. Some data regarding index procedure details, including the number of stents used and the type of target vessel for revascularization, which can potentially affect the future need for revascularization, were not obtained and hence can affect the outcomes of the study. This calls for more detailed data collection for cancer patients in large-scale PCI registries to further validate the findings of our study.

## Conclusion

In conclusion, cancer patients with CAD treated with BMS had similar overall survival and need for revascularizations compared to patients treated with DES. Our study revealed no increased risk of stent thrombosis or restenosis as well as all-cause mortality in cancer patients when comparing BMS vs. DES. As cancer therapy continues to evolve, the survival of these patients is expected to increase. Hence, greater use of DES may benefit these patients over a longer follow-up period. As such, the choice of stents in these patients should factor in the stage of cancer, expectant survival, and overall prognosis.

## Data availability statement

The raw data supporting the conclusions of this article will be made available by the authors, without undue reservation.

## Ethics statement

The studies involving human participants were reviewed and approved by the MD Anderson Cancer Center Institutional Review Board. Written informed consent for participation was not required for this study in accordance with the national legislation and the institutional requirements.

## Author contributions

TA contributed to conception or design of the work, acquisition, analysis, or interpretation of data for the work, and drafting the work or revising it critically for important intellectual content. HP and AA contributed to the acquisition, analysis, or interpretation of data for the work, and drafting the work. EK contributed to revising it critically for important intellectual content. JS did analysis of the data and provided approval for publication of the content. KC, KM, KB, MC, and CG contributed to revising it critically for important intellectual content and final approval. CI contributed to revising it critically, provided final approval of publication, agreed to be accountable for all aspects of the work in ensuring that questions related to the accuracy or integrity of any part of the work are appropriately investigated, and resolved. All authors contributed to the article and approved the submitted version.

## Funding

The statistical analysis work was supported in part by cancer center support Grant No. 24 (NCI Grant No. PA30 CA016672).

## Conflict of interest

The authors declare that the research was conducted in the absence of any commercial or financial relationships that could be construed as a potential conflict of interest.

## Publisher's note

All claims expressed in this article are solely those of the authors and do not necessarily represent those of their affiliated organizations, or those of the publisher, the editors and the reviewers. Any product that may be evaluated in this article, or claim that may be made by its manufacturer, is not guaranteed or endorsed by the publisher.
